# Optimising a clinical decision support tool to improve chronic kidney disease management in general practice

**DOI:** 10.1186/s12875-024-02470-w

**Published:** 2024-06-19

**Authors:** Barbara Hunter, Sandra Davidson, Natalie Lumsden, Sophie Chima, Javiera Martinez Gutierrez, Jon Emery, Craig Nelson, Jo-Anne Manski-Nankervis

**Affiliations:** 1 Department of General Practice and Primary Care, University of Melbourne, Melbourne, Australia; 2 Western Health Chronic Disease Alliance, Western Health Melbourne, Melbourne, Australia; 3 Centre for Cancer Research, University of Melbourne, Melbourne, Australia; 4 The Primary Care Unit, University of Cambridge, Cambridge, UK; 5 Family Medicine Department, School of Medicine, Pontificia Universidad Católica de Chile, Santiago, Chile; 6 Department of Medicine – Western Health, University of Melbourne, Melbourne, Australia

**Keywords:** Chronic disease, Chronic kidney disease, Cardiovascular, General practice, Primary care, Patient care, Health technology

## Abstract

**Background:**

Early identification and treatment of chronic disease is associated with better clinical outcomes, lower costs, and reduced hospitalisation. Primary care is ideally placed to identify patients at risk of, or in the early stages of, chronic disease and to implement prevention and early intervention measures. This paper evaluates the implementation of a technological intervention called Future Health Today that integrates with general practice EMRs to (1) identify patients at-risk of, or with undiagnosed or untreated, chronic kidney disease (CKD), and (2) provide guideline concordant recommendations for patient care. The evaluation aimed to identify the barriers and facilitators to successful implementation.

**Methods:**

Future Health Today was implemented in 12 general practices in Victoria, Australia. Fifty-two interviews with 30 practice staff were undertaken between July 2020 and April 2021. Practice characteristics were collected directly from practices via survey. Data were analysed using inductive and deductive qualitative analysis strategies, using Clinical Performance - Feedback Intervention Theory (CP-FIT) for theoretical guidance.

**Results:**

Future Health Today was acceptable, user friendly and useful to general practice staff, and supported clinical performance improvement in the identification and management of chronic kidney disease. CP-FIT variables supporting use of FHT included the simplicity of design and delivery of actionable feedback via FHT, good fit within existing workflow, strong engagement with practices and positive attitudes toward FHT. Context variables provided the main barriers to use and were largely situated in the external context of practices (including pressures arising from the COVID-19 pandemic) and technical glitches impacting installation and early use. Participants primarily utilised the point of care prompt rather than the patient management dashboard due to its continued presence, and immediacy and relevance of the recommendations on the prompt, suggesting mechanisms of compatibility, complexity, actionability and credibility influenced use. Most practices continued using FHT after the evaluation phase was complete.

**Conclusions:**

This study demonstrates that FHT is a useful and acceptable software platform that provides direct support to general practice in identifying and managing patients with CKD. Further research is underway to explore the effectiveness of FHT, and to expand the conditions on the platform.

**Supplementary Information:**

The online version contains supplementary material available at 10.1186/s12875-024-02470-w.

## Background

Chronic disease affects 47% of the Australia population and accounts for 52% of all hospitalisations [1]. Primary care is ideally placed to identify patients at-risk or in the early stages of chronic disease and to implement prevention and early intervention measures that improve clinical outcomes and reduce hospital admissions [2]. For example, timely treatment of chronic kidney disease (CKD), which affects almost a million Australians and is associated with 17% of all hospitalisations [3], can reduce deterioration of kidney function by up to 50%, which in turn reduces the likelihood that patients will progress to end-stage CKD and require kidney replacement therapy [4]. However, studies show that primary care is missing opportunities to detect and treat chronic disease. Khanam et al. [2] found that only 20% of primary care patients with laboratory evidence of stage-3 kidney disease had kidney disease documented in their medical record and only 25% were monitored in line with the recommendations from Kidney Health Australia (i.e., annual monitoring of blood pressure, urine albumin-to-creatinine ratio, estimated glomerular filtration rate and serum lipids).

Evidence suggests that technological interventions that include audit, feedback, and decision support functions can improve the identification and management of chronic disease in primary care [5–9]. Key barriers to successful implementation include poor fit with existing workflow, limitations in clinical applicability to patients with multimorbidity and overly simplistic systems [10]. 

Future Health Today (FHT) is a co-designed technological intervention that aims to identify primary care patients at risk of, or with undiagnosed or untreated chronic disease, and provide practitioners with guideline concordant recommendations for patient care [11]. The FHT software is integrated with the electronic medical record (EMR) of the primary care practice and uses sophisticated algorithms to read EMR data and identify patients with indicators of chronic disease or disease risk. At this time, FHT has been programmed to integrate with three commonly used Australian EMR software systems (Medical Director [12], Best Practice [13] or ZedMed [14]).

In 2019 a prototype of FHT was piloted in two general practices to test its functionality and feasibility [15]. In 2020, FHT was implemented in 12 general practices across Victoria, with the intention of optimising the tool and implementation processes. The implementation was evaluated to identify the barriers and facilitators to successfully integrating FHT in these practices using the Clinical Performance - Feedback Intervention Theory (CP-FIT; 16). The CP-FIT framework draws together numerous analytical frameworks to examine the effectiveness of tools designed to improve clinical feedback and provides a realist lens through which to understand implementation in real-world situations. In this report, we describe how implementation of FHT in practices using three different EMR platforms was analysed and improved using the CP-FIT framework. Although FHT was expanded towards the end of the intervention period to include other chronic conditions such as cancer [16] and cardiovascular disease, this paper focuses on the implementation as it relates to the first suite of recommendations implemented in FHT: the CKD recommendations.

## Methods

We undertook a qualitative evaluation of the implementation of FHT in 12 primary care practices in Victoria, Australia, underpinned by an action research philosophy [17–19] focused on continuous improvement. Data analysis was undertaken throughout the project to inform ongoing improvements in the implementation and technology, and participants had the opportunity to reflect on adjustments made as a direct result of their feedback [20–23]. The study received ethics approval from the Human Research Ethics Committee at the University of Melbourne (ID:1953614).

### The intervention

FHT software extracts data from a general practice’s EMR each night and processes the data using evidence-based algorithms to identify patients who may benefit from implementation of guidelines for diagnosis or management of a target condition. The results are made available to general practice staff through a point-of-care (POC) prompt and web-based ‘dashboard’. Supplementary file 1 provides screenshots of the tool. The POC provides an individualised prompt when a patient EMR file is opened by a staff member who has permission to access patient information. FHT appears as a small box on the user’s screen with recommendations for patient care. Each recommendation links to the relevant guidelines and resources (including consumer resources). Users can close or minimise the POC box.

The dashboard requires users to login to a practice-specific, password protected web address. Once opened, the dashboard lists patients who meet criteria for follow-up and records whether they are being managed according to the FHT recommendations. It provides educational videos and documents and links to peak-body web-hosted clinical guidelines and consumer resources. No patient information is sent outside the practice. FHT sends an automatic nightly summary report to the FHT team providing an aggregate count of patients identified by each algorithm. This is used for evaluation purposes and to ensure that the algorithms are running correctly in practices.

FHT was installed with CKD algorithms and recommendations (see Table 1) in 12 general practices between May and September 2020. After installation, practices were encouraged to use FHT, with the request that at least one general practitioner (GP) and general practice nurse (GPN) engage with the platform. Each practice was offered online live training sessions on how to use FHT.

**Table 1:** CKD recommendations

Recommendation	Clinical Indicator
Kidney Health Check recommended (eGFR, ACR, BP).	Patients without a diagnosis of CKD and • who have one or more risk factors and • without a kidney health check
Possible diagnosis of CKD: confirmatory testing recommended.	Patients without a diagnosis of CKD and • who have an abnormal eGFR or ACR test result
Pathology test consistent with CKD - review and code diagnosis.	Patients without a diagnosis of CKD and • who have an abnormal eGFR or ACR test result and • who have previous abnormal eGFR or ACR results that together with the current result indicates the patient has CKD
Consider Initiation of ACEI or ARB for CKD management	Patients with a CKD diagnosis and • who are not on an ACEI or ARB and • BP not at target and • who have clinical results that indicate the patient BP potentially should be managed on ACEI/ARB
Consider initiation of ACEI or ARB (note: BP in target).	Patients with a CKD diagnosis and • who are not on an ACEI or ARB and • BP at target and • who have Hypertension or High Risk of CVD
Consider Intensification of ACEI or ARB dosage or initiation of another agent. Other option: Elevated blood pressure despite treatment - consider review.	Patients with a CKD diagnosis and • who are already on an ACEI or ARB and • BP not at target
Consider Initiation of statin.	Patients with a CKD diagnosis and • who are not on a Statin and • who have a high risk of CVD (a Framingham risk score of > 15% or are automatically at high risk)
Consider Intensification of Lipid-lowering Therapy.	Patients with a CKD diagnosis and • who are already on a Statin and • whose lipids (total cholesterol, LDL, TG) are not at target

Acronyms:

eGFR (Estimated Glomerular Filtration Rate); ACR (Albumin-to-creatinine ratio); BP (blood pressure); ACEI (Angiotensin converting enzyme inhibitors); ARB (angiotensin receptor blockers); CKD (chronic kidney disease); CVD (cardiovascular disease); LDL (Low density lipoprotein); TG (Triglyceride)

### Participants and setting

A purposive sample of six general practices in metropolitan Melbourne and six in rural/regional Victoria (*N* = 12) were recruited through VicREN, the University of Melbourne’s practice-based research and education network [24, 25] via newsletter advertisements and direct email and phone contact. Practices were eligible to participate if they used Medical Director [12], Best Practice [13] or ZedMed [14] EMR software. Twenty-one eligible practices expressed initial interest in the project. Twelve practices were selected from this group, ensuring even spread across metropolitan and regional locations. The 12 practices were treated as separate sites for implementation however two practices were owned by a single entity, shared staff across both sites and shared an EMR database, and were regarded as a single site for analytic purposes. Practices were remunerated for their participation in the project and individuals participating in interviews were provided with vouchers.

Each practice nominated a staff member to be a FHT champion. Champions undertook software training, a practice assessment survey and were asked to participate in two interviews. Champions identified staff who were interested in participating in an interview/focus group about FHT, with a target of three participants per practice. This target was selected based on the average size of practices and anticipated response rates of practice staff, but was flexible to ensure that sufficient diversity and conceptual depth [26] was achieved.

### Data collection

Practice champions completed a practice assessment survey capturing practice characteristics (including number of patients, practice staff, full-time and part-time employees; staff demographics; and previous research and quality improvement activities). Semi-structured interviews informed by CP-FIT (see Supplementary file 2 for interview guide) were conducted by BH and SC. Interviews were conducted via phone and audio-recorded. It was intended that interviews would take place one month(T1) and two months after installation(T2) and a focus group would be held three months after installation(T3). However, technical and COVID-19 related disruptions meant that T1 interviews occurred 2–6 months after installation, T2 interviews occurred 1–4 months after T1 interviews and focus groups were replaced with targeted interviews. T1 interviews assessed usability of FHT, including frequency of use, components used, complexity of tool, fit with usual workflow and requests for change to the platform. T2 explored the impact of using FHT on daily practice, including how it is used in consultations, integration across the organisation, perceived usefulness, impact on individual patient care, and requests for changes. T3 focused on use of specific components or recommendations displayed on the platform. The original study design also included 30 patient interviews which were ultimately abandoned due to pandemic-related recruitment complexities.

### Analysis

**Fig. 1:**
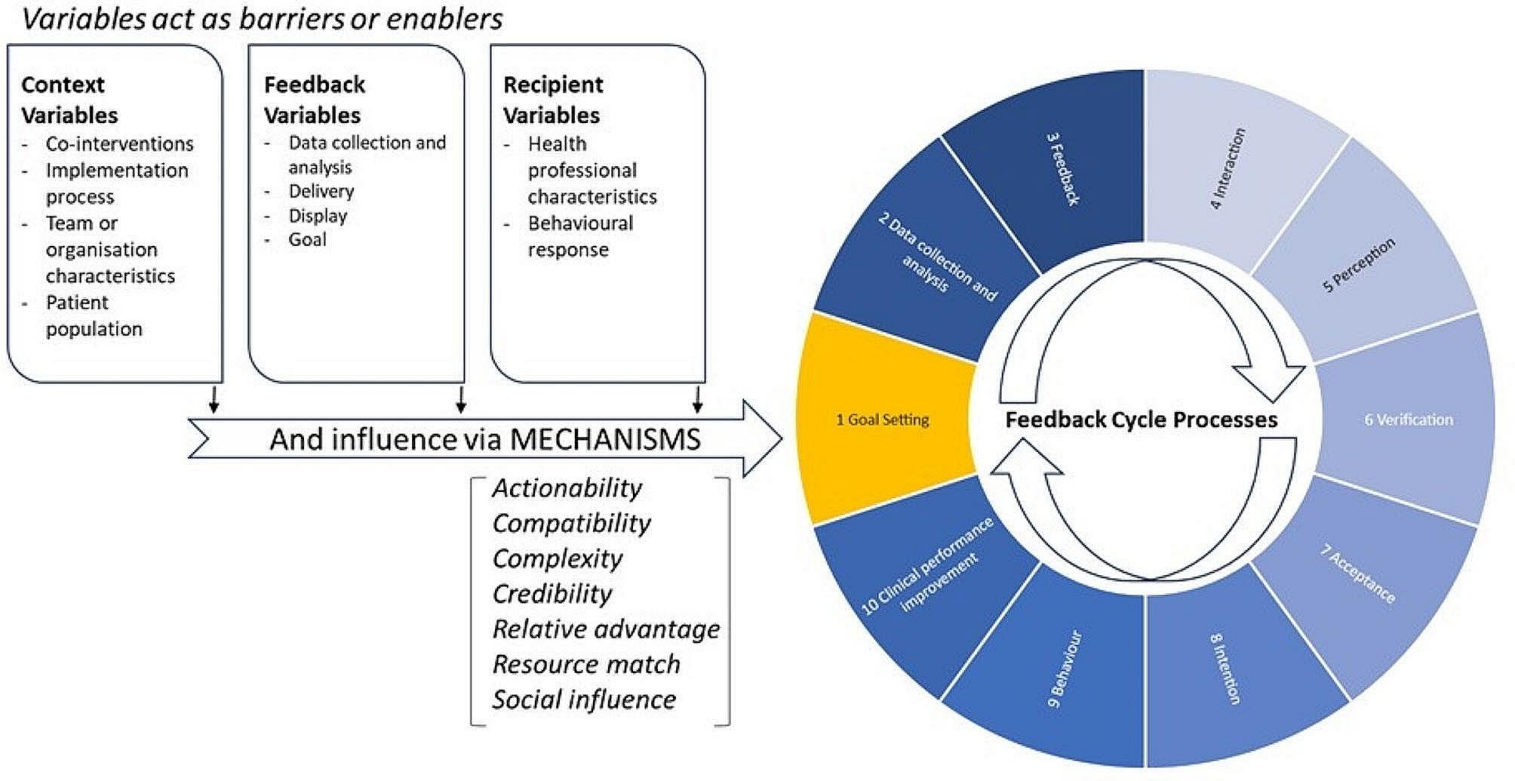
Clinical performance feedback intervention theory (CP-FIT) [28]

Survey data were analysed using simple descriptive statistics. Interview recordings were transcribed by a professional transcription company and then reviewed by a researcher (BH) prior to uploading data to NVIVO [27], where they were analysed by two researchers (BH&SC) using inductive and deductive analysis. Analysis was conducted iteratively over the course of the project, with interviews informing implementation and changes reviewed in subsequent interviews. CP-FIT [28] provided the analytic framework. CP-FIT was developed to identify the mechanisms underpinning an effective feedback intervention and can be used in a process evaluation to identify barriers and facilitators to the successful implementation of an intervention (see Fig. 1). CP-FIT surmises that effective feedback is a cyclical process of goal setting, data collection and analysis, feedback delivery, recipient interaction, perception and acceptance of feedback, intention, behaviour, and clinical performance improvement. The effectiveness of feedback is reduced if any element in the cycle is disrupted.

FHT provided direct access to the feedback cycle through its clinical decision support (POC) and audit functionalities (dashboard). This evaluation explored the variables that influence how the feedback cycle is accessed including: context variables (comprising characteristics of the team, organisation, patient population, co-interventions, and the implementation process); recipient variables (comprising what participants bring with them regarding knowledge and attitudes and how they respond to the intervention); and feedback variables (comprising the intervention itself).

## Results

FHT champions were GPNs(*n* = 4), GPs(*n* = 3) and general practice managers (GPMs; *n* = 5). In total, 52 telephone interviews with 30 individuals were completed between July 2020 and April 2021: forty-six interviews had one participant, three interviews had two participants, and three interviews had three participants. Of the 30 individuals participating, seven participants took part in one interview, 18 participated in two interviews and five participated in three interviews. Interviews ranged in duration from 15 to 30 min.

Table 2 provides an overview of the key characteristics of the participating practices. Number of patients registered with practices ranged from 12,059 to 91,781. The largest practice employed 46 staff, including 24 GPs and 10 GPNs, while the smallest practice employed seven staff, including four GPs and no GPNs. Practice EMR operating systems included Medical Director(*n* = 5), Best Practice(*n* = 4) and ZedMed(*n* = 3). All practices had previously undertaken quality improvement activities and been involved in research or teaching activities with the University of Melbourne. One metropolitan practice and one regional practice had previously used an early prototype of FHT [15], and six had been involved in co-design [11]. Nine of the twelve practices elected to continue using FHT after the study ended.

**Table 2:** Setting

	MetroPrac1	MetroPrac2	MetroPrac3	MetroPrac4	MetroPrac5	MetroPrac6	RegPrac1	RegPrac2	RegPrac3/4	RegPrac5	RegPrac6
*# Patients*	60,907	71,829	27,663	23,346	21,381	12,059	13,627	38,418	16,595	91,781	27,799
*Total Staff*	-	32	9	13	7	-	30	20	21	46	21
*- PT N(%)*	-	20 (63%)	7 (78%)	8 (62%)	4 (57%)	-	16 (53%)	16 (80%)	12 (57%)	24 (52%)	11 (52%)
*- FT N(%)*	-	11 (34%)	2 (22%)	5 (38%)	3 (43%)	-	14 (47%)	4 (20%)	9 (43%)	22 (48%)	10 (48%)
*# GP/GPR*	-	16 (10PT, 6FT)	4 (3 PT, 1FT)	6 (3PT, 3FT)	4 (2PT, 2 FT)	-	10 (4PT, 6FT)	6 (3PT, 3FT)	6 (6PT)	24 (16PT, 8FT)	10 (8PT, 2FT)
*# GPNs*	-	3 (2PT, 1FT)	1 (PT)	1 (FT)	0	-	5 (3PT, 2FT)	4 (PT)	4 (3PT, 1FT)	10 (FT)	3 (2 PT, 1FT)
*# GPM*	-	1	1	1	1	-	1	1	1	1	1
*# Business manager*	-	2	0	0	0	-	1	1	0	1	0
*#POC installed*	17	2	1	1	3	1	32	9	11	1	7
*# staff reporting using FHT*	-	3	5	3	1	-	2	2	4	1	2
*Operating system*	ZedMed	ZedMed	Medical Director	Medical Director	Medical Director	Best Practice	Best Practice	Best Practice	Medical Director	ZedMed	Best Practice
*Installation structure*	Individual	Network	Network	Network	Individual	Network	Individual	Individual	Hybrid	Network	Individual
*Installation date*	May 2020	Jun 2020	Aug 2020 (testing)	Sept 2020	Aug 2020	Aug 2020	May 2020	May 2020	Sept 2020	June 2020	May 2020
*Reported use start*	Late 2020	Sept 2020	Oct 2020	Sept 2020	Sept 2020	Sept 2020	June 2020	July 2020	Oct 2020	June 2020	Jun 2020
*Use of FHT*	Limited users, limited use	1–3 GPs + GPN, POC only	Practice wide use, POC only	1–3 GPs + GPN, POC only	Limited users, limited use	1–3 GPs plus GPN, POC & D/b	1–3 GPs + GPN, POC only	1–3 GPs + GPN, POC only	Practice wide use, POC & D/b	Limited users, limited use	1–3 GPs plus GPN, POC & D/b
*Previous FHT involvement*	Yes – co-design and pilot site	Yes – co-design	Yes – co-design	No	No	Yes – co-design	Yes – co-design and pilot site	Yes – co-design	No	No	No

Notes:

1. RegPrac3 and RegPrac4 share a server and have significant staff overlap. They are treated as a single site throughout the evaluation

2. MetroPrac1 and MetroPrac6 did not supply information via the practice assessment survey

### Did FHT facilitate a ‘feedback cycle’?

Interviewees generally reported that FHT was clinically meaningful in their daily practice and was associated with beneficial clinical outcomes. Several interviewees noted the importance of a specific focus on CKD and appreciated prompts (Table 3, Quote 1), and noted the presence of the tool facilitated better patient care (Table 3, Quote 2). One participant suggested that the tool could assist with additional training and support for medical students and GP registrars (Table 3, Quote 3). Participants reported outcomes related to the CKD module that suggest FHT facilitated movement through a feedback cycle and improved clinical performance, with outcomes including:

**Table 3:** CP-FIT Themes and associated quotes

	Component	Quote	Participant
1	Feedback cycle – performance improvement	“I still think like a Future Health person now; that’s what’s really changed… like I keep thinking oh what’s their renal function, have you done their - you know it’s amazing. I love it. …I’m a total convert.”	MetroPrac6_GP1_F
2	Feedback cycle – goal setting	“…the more routine care and planning, it just gets lost in the acute medicine, which is why I’m interested in FHT…Oh, it goes beyond useful. I think not only is it useful, I think it should be mandatory. It’s like – it’s the equivalent of the warnings that we get when we are prescribing.”	RegPrac4_GP1_M
3	Feedback cycle – goal setting	“I do quite a lot of medical student teaching and we did think just over the past few weeks that it would be really useful for the medical students to look at Future Health Today prompts and to speak to the patient specifically about these prompts so that that would be a - it wouldn’t be the nitty-gritty of the consultation. It would be half an hour or so that the patient would be able to spend thinking about a particular problem or a particular issue that was coming up.”	RegPrac2_GP2_F
4	Feedback variable – display	On some days, I may really skip it and some days I just check it for every single patient.	MetroPrac3_GP2_F
5	Feedback variable - display	“I like that it’s just on the side and prompts if it’s a different colour to tell me to review. I do like just it’s a subtle -it’s not in your face that’s coming up all the time as a reminder, because we’ve got so many flashes.”	MetroPrac6_GP3_F
6	Feedback variable – display	“…the shorter it is, the more likely people are to read them, for sure.”	RegPrac4_GP1_M
7	Feedback variable – delivery	“…I think the front story should be short and smart but I think you should be able to click on the back story and be able to get some evidence or guidelines.”	RegPrac2_GP2_F
8	Feedback variable – accuracy	So, it can’t always read data, so sometimes it gives you suggestions that are not relevant and that obviously takes time to look and go, no, I have done that and therefore that’s annoying. When it’s told me something I’ve not thought about … that’s good. But when it tells you things you’ve already done, that’s quite irritating and you’re less likely to notice it the next time.	RegPrac2_GP2_F
9	Feedback cycle – performance improvement	“Basically I love it and it means that I reduce dosage and things. I just use it in a clinical sense, I think it’s fantastic. It makes me look at drug doses, it makes me discuss it with the patients, it probably means I prescribe more statins than I have before, I think it’s really changed my behaviour. I’ve been more aggressive with my blood pressure lowering. I just think it’s made me [a] better doctor really.”	MetroPrac6_GP1_F
10	Context variable - reminders Feedback variable - goal	“I thought that the whole point of the Future Health Today was a point of care thing that just came up to remind me of some of the things they should be doing.”	RegPrac1_GP2_F
11	Context variable – teamwork	“I’ve been looking at the dashboard to try to – I’ve been basically building different cohorts, that once I get the okay from people we will start recalling.”	MetroPrac1_GPN_F
12	Context variable – workflow	Bottom right-hand corner of the screen, hit on that and have a quick look and often you can deal with it -probably three quarters of the patients you can deal with it straight away. There’re a few others that it’s got an enormous amount of detail and invites another session with the patient.	MetroPrac5_GP1_M
13	Context variable – conflicting priorities	… it’s simple, it’s not complicated. You don’t really have to think about it, you just have to open up the thing and do what it tells you to do. All you need is 30 s, but you know what it’s like when you’re busy, 30 s seems like a big impost.	MetroPrac2_GP1_M
14	Recipient variables – knowledge etc. Context variable - workflow	“… the one came up recently was new. I think, my golly – so I actually had to look up the guidelines and I thought that was too awkward because it was disruptive in the practice. I thought, oh, this sounds a little bit like it’s not appropriate and I really was taken aback… Having that popping up … took me offside because it was new. I thought, mm. Because FHT … was meant to prompt but not to interfere with the thought processes too much so you could delay it, defer it.”	MetroPrac3_GP1_M
15	Recipient variables – knowledge etc. Context variable - workflow	there was another thing on it that I didn’t understand; this patient could be eligible for the (project). I didn’t know what that was and I haven’t time to look it up. So I thought, oh, that’s annoying because I don’t know why that is there.”	RegPrac2_GP2_F
n/a	Feedback variable – accuracy, evidence base Recipient variable – knowledge	“This is just data, and this is what might indicate such and such and this is what you might do about it. It’s like spoon feeding, but in one way it’s supporting their decisions. Otherwise, you need to run an educational module separate to that to say if this prompt comes up, you need to watch this YouTube video that’s educational about it. That type of idea. Do you need more information? Do you understand what this means? If not, click here and it will load a link to a browser or whatever.”	MetroPrac3_GP1_M
n/a	Recipient variable – knowledge	when asked if they would act on the platelet recommendation: “Well, it’ll – in my case I’d probably just shelve it and then I’d go through and work out what I would do. I had the same discussion last night talking about the importance of journal searching and supporting information gathering.”	MetroPrac3_GP1_M
n/a	Feedback variable – accuracy Context variable – workflow, competing priorities	“Seeing a prompt there, I go, oh, wow look at that, and then – say if it’s for CKD, I’d actually go through and make sure it was true. Make sure that is a real reading because as you know it doesn’t always tell you exactly what reality is. It might have been just a dehydration reading. Yeah, so – and then I think well, okay, park that. Put that in the list of things I’m going talk about in the file, so just make some notes. Then if time allows, I’ll deal with it. It won’t be the first thing because when the patient comes in, they’re coming in because they want to come in.”	MetroPrac3_GP1_M

- Ordering tests they may otherwise not have ordered.
- Remind GPs of things they otherwise may forget (e.g., providing smoking cessation information).
- Improving record keeping in the patient record (e.g., correct diagnosis entered in the correct EMR field).
- Improving knowledge and awareness of CKD and applying that knowledge to future patients, including where FHT did not provide a prompt.

### Feedback variables were most likely to enhance or impede use of FHT

**Fig. 2:**
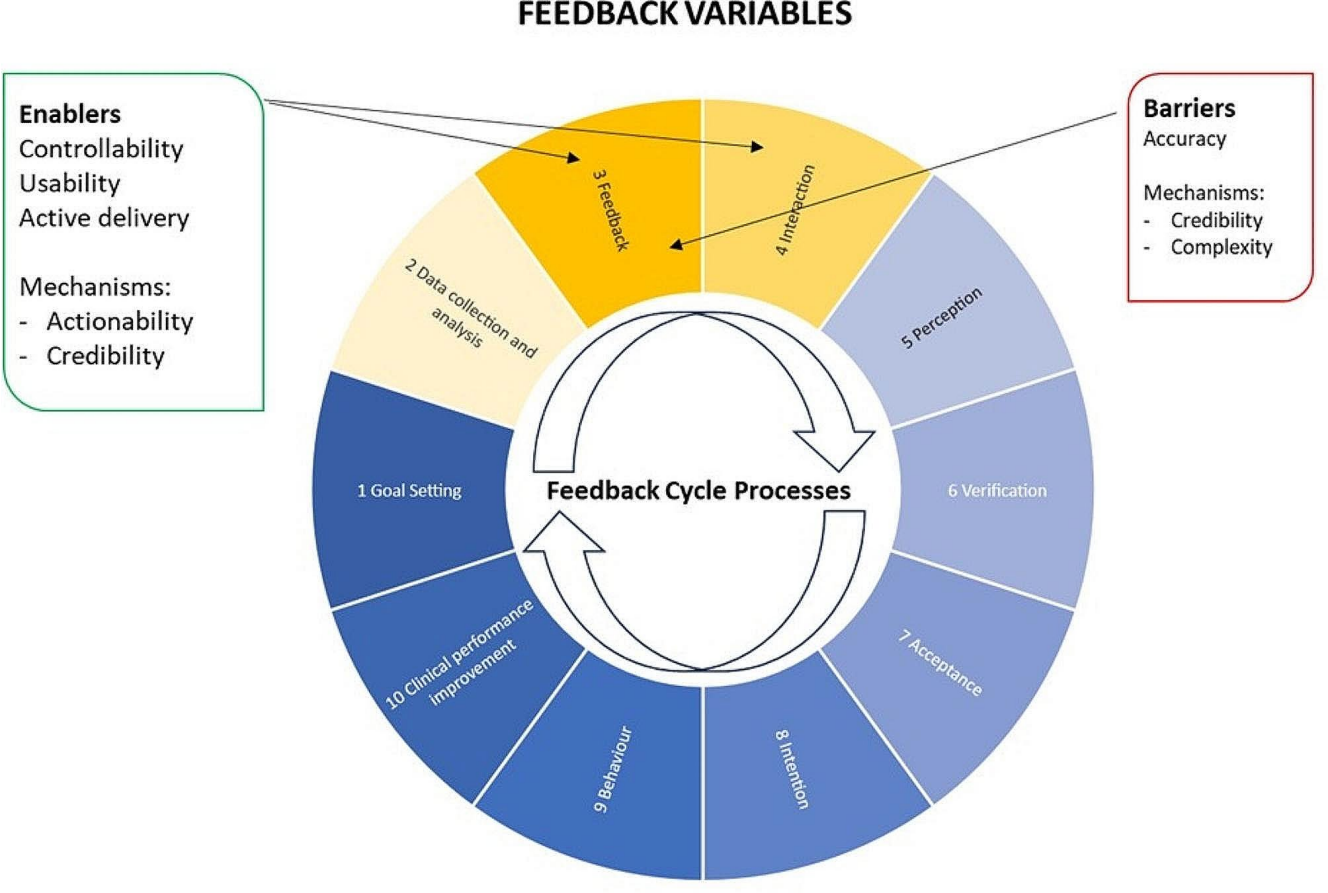
Feedback variables influencing use of FHT

Variables relating to the design and delivery of feedback via FHT had the greatest impact on use of the tool, acting as both a facilitator and barrier (see Fig. 2). The way the information was displayed was critical (Step 3 of the feedback cycle) and determined whether participants remained engaged in the feedback cycle (Step 4 of the feedback cycle). Despite the initial concerns of some GPMs and GPNs, the POC prompt was not considered annoying by most interviewees, rather they reported it being useful, unobtrusive, and easy to either engage with or ignore as required (Table 3, Quote 4). Participants indicated that the prompt was so unobtrusive they were concerned that they may start to ‘*not see it’* over time. Users appreciated the placement and colour of the prompt (Table 3, Quote 5), with the minimised view of FHT considered to be useful to trigger attention (minimised green = no recommendation; minimised orange = active recommendation) and enhance usability. This POC prompt was used more frequently than the dashboard, as discussed further below (context variables).

Also important was the way the information was delivered, with the need for short and actionable recommendations (Table 3, Quote 6) and the opportunity to delve further into guidelines or resources as required (Table 3, Quote 7). Recommendations for a specific action, written in simple and clear language, were positively received and illustrated the importance of the participant feeling able to respond to the feedback (controllability). Recommendations that involved a complex action and/or used non-specific language were less favourably received. For example, the recommendation for lipid lowering therapy (Table 1) was misinterpreted as relating only to the introduction or optimisation of medications, rather than also applying to non-medicinal strategies.

A significant barrier identified in the early phase of implementation was the accuracy of the patient lists generated by the algorithms and the recommendations attached to the POC and dashboard (step 2 of the feedback cycle). On some occasions, feedback indicated that clinicians felt they had already addressed the recommendation identified by FHT. This was the result of occasional poor quality or missing data in the practice EMR and/or the way FHT filtered information. Irrespective of the source of the error, repeated interactions of this type were likely to reduce use of the tool (Table 3, Quote 8). There was a clear reduction in use among clinicians who questioned the accuracy of the FHT output, with one practice (RegPrac5) ceasing use of FHT altogether, despite education and further changes to the clarity of algorithms. However, more commonly, once users were satisfied that the lists and recommendations were accurate, experiences with using FHT and beliefs about its actionability were very positive. One GP who stopped using FHT because of its inaccuracies later re-engaged with the updated version and found it to be a useful clinical support (Table 3, Quote 9) and facilitator to self-reported improved clinical performance.

These feedback variables illustrated the interplay of mechanisms relating to the actionability, credibility and complexity in facilitating trust with clinicians, and the importance of honest engagement with end users (clinicians) to review data quality and improve the functioning of the tool.

### Context variables created complex challenges

**Fig. 3:**
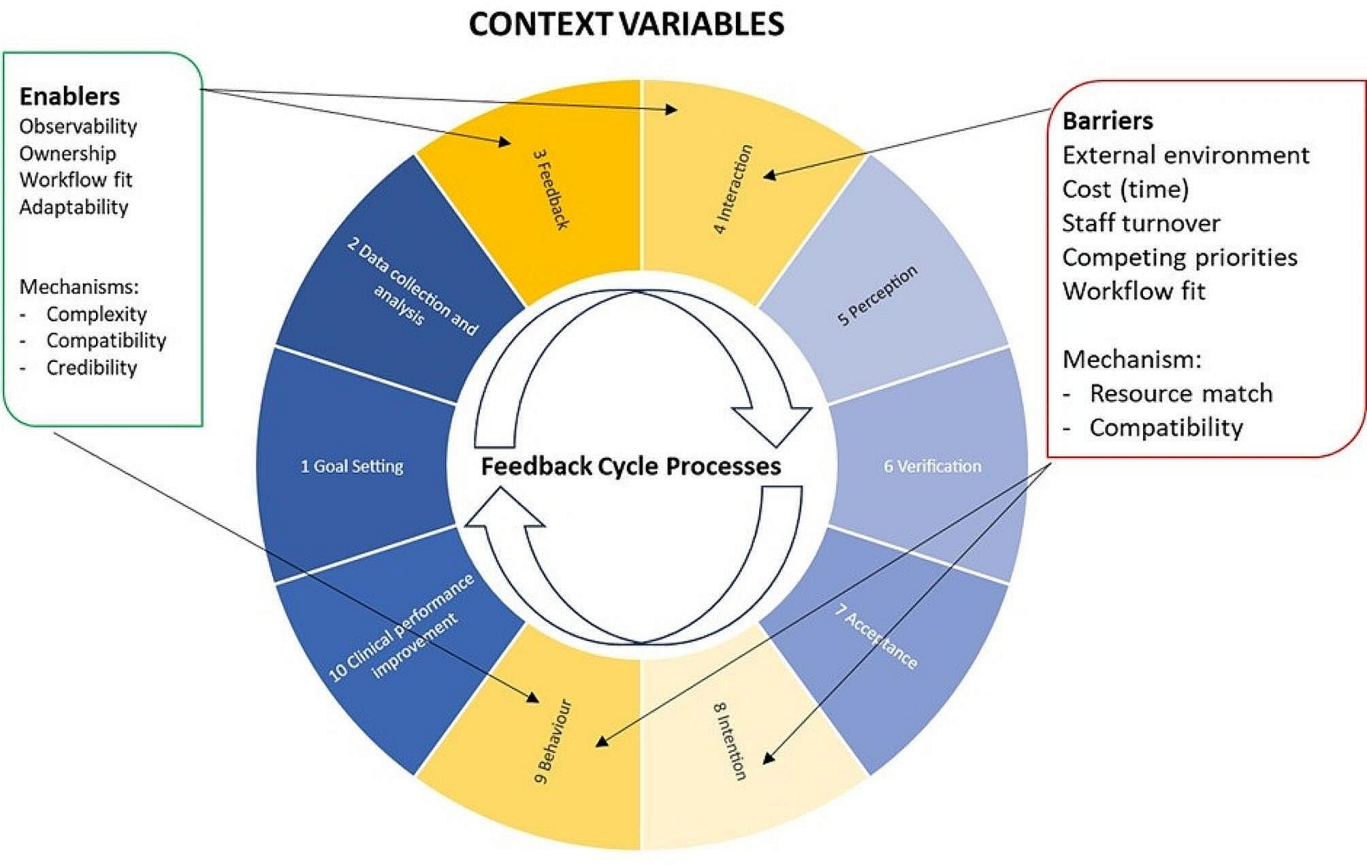
Context variables influencing use of FHT

Context variables relating to organisational and team characteristics and the implementation process acted as both facilitators and barriers for use of FHT (see Fig. 3). However, use was negatively impacted by context variables related to the external environment (e.g., pandemic, pandemic vaccination readiness and installation of technology). Pandemic-related pressures delayed the commencement of the project and influenced practices’ emotional and cognitive space available for engagement with a new initiative. Staff absences/turnover (illness and resignations), multiple lockdowns, telehealth, reduced patient attendance, implementation of COVID-19 testing, and preparedness for vaccinations reduced the time and resources available to implement FHT.

Despite this, each practice engaged with FHT, both in the initial installation phase and throughout the implementation period. Some installations went smoothly, however there were challenges associated with the installation of FHT across the different sites and operating systems, including: insufficient IT support in the practice; practice-based virus protection software blocking FHT; auto-updaters not updating FHT algorithms; POC box disappearing; practice system updates causing glitches with FHT; algorithms not reading EMR correctly or quirks in EMR systems impacting algorithm runs; practices requiring POC to be installed on each machine rather than on a single network server; issues with dashboard functionality and display (e.g., data filtering and exporting issues); and problems with passwords (i.e., reset every three months; difficulty resetting passwords). These issues were related to the practice-specific environments, not the EMR operating system utilised. One practice also believed that FHT negatively affected the upload ability of their central processing unit (CPU) and subsequently decommissioned FHT. However, after an investigation by the technical support team and IT provider could not identify any issues with the CPU, the practice reinstalled FHT. The responsiveness of the FHT team to issues raised in practices increased engagement and enhanced a feeling of shared ownership of FHT.

Whilst these challenges interfered with practices’ ability to interact with FHT (Steps 3&4 of the feedback cycle), once the technical challenges had been addressed use of the POC function became routine in most practices (Table 2). Most practices utilised the POC prompt only (Table 3, Quote 10), and 10 practices indicated that not all staff used FHT, either due to lack of access (e.g., FHT not installed on their computer in the practice or computer used to remotely access the practice network) or lack of interest (e.g., ignoring or closing the POC prompt when it appears). GPs frequently used only the POC and GPNs reported using it to complete care plans. It was used both in, and in preparation for, consultations.

In contrast, dashboard use was limited, typically shortly after FHT was installed and then rarely used again unless prompted by the FHT team. GPNs and GPMs were the most likely to use the dashboard and the level of use depended on both time and GPs engagement in proactive recall and management of patients. Those who accessed the dashboard did so to explore its capability (Table 3, Quote 11), but encountered both technological blockers (e.g., refining and filtering functionality not working as expected; export output omitting criteria or providing too much information) and organisational blockers (e.g., difficulties in accessing staff to work with them on recall activities; lack of time for recall activities).

Those who used FHT most extensively actively commenced use within the first month post-installation, whilst those who used it least had a longer time-lag between installation and first use. Delays in first use resulted from lack of time, COVID-19 impacts on the practice and perceptions of the importance of the tool (i.e., not a high priority). The most common starting point for integrating FHT into a practice was for a small number of ‘key users’ to engage with the platform, usually the GPN and a GP. In one practice the GPM took primary responsibility for investigating the usefulness and functionality of the platform, before including GPs and GPNs.

Interviewees reported that using the POC occurred naturally within the workflow of daily practice. Staff interacted with the POC when it appeared on their screen, either by acknowledging and acting on the recommendation, acknowledging and not acting on the recommendation, actively minimising or closing the box, or ignoring it completely. The main challenge to acting on the FHT recommendations was lack of time or competing patient concerns (Table 3, Quotes 12&13). In contrast, the time and effort required to move through multiple steps to access the dashboard was a disincentive for most participants. The cognitive effort associated with remembering/finding the web address and login was not counterbalanced by the usefulness of the information provided on the dashboard and was perceived to add little value to current practice.

External context variables had the most significant impact on practices’ capacity to engage with FHT, with mechanisms relating to the mismatch of resources driving a reduction in engagement. Compatibility of tools with workflow, workload and practice technical set-up acted as a barrier for some practices using FHT, however when mitigated by the credibility and lack of complexity of the tool, it acted to increase use of FHT.

### Recipient variables influenced by time and exposure

**Fig. 4:**
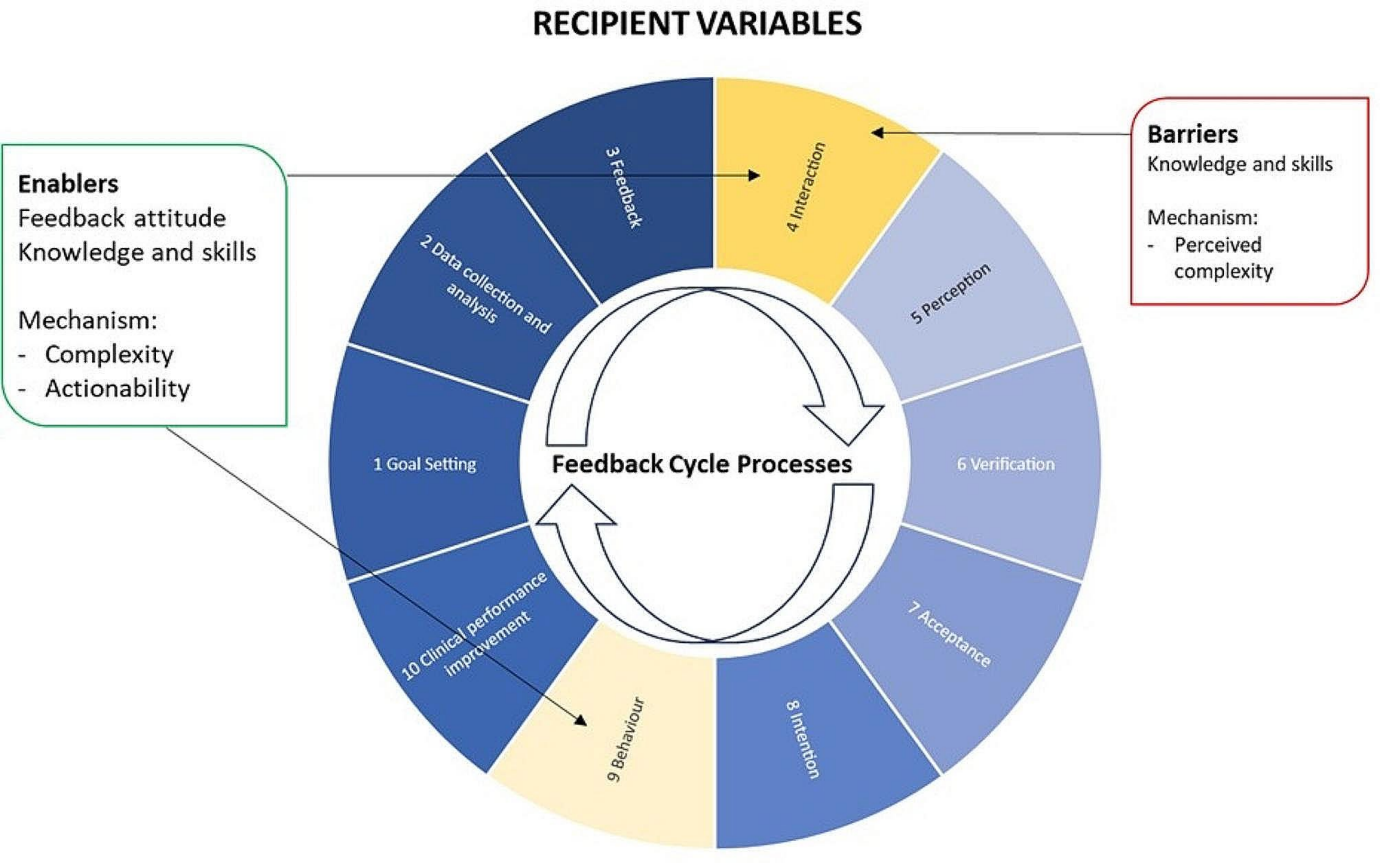
Recipient variables influencing use of FHT

Recipient variables (comprising what our participants bring with them regarding knowledge and attitudes and how they respond to the intervention) had a greater impact on participants sense of how well they were using FHT than was reflected in their actual use. When asked to describe use, it was clear participants were using it effectively and were not hitting significant barriers to use. In fact, it appears that despite the evolution of participant perspectives on FHT as they gained confidence in use and increased their clinical awareness of CKD, actual use was not largely impacted by recipient variables (see Fig. 4). If anything, curiosity to learn more about CKD and improve their clinical performance facilitated continued use, with lack of complexity and ease of actioning recommendations acting as mediating mechanisms. Participants adjusted their perspective on the usability and usefulness of FHT over the study period. These changes were influenced by use of the platform itself, but also related to the external contextual space in which participants were situated (namely, the pandemic and its impacts on general practice). Interviews indicated that some staff had pre-conceived perceptions that using FHT would be complicated, difficult to use, and that there must be ‘more to it’, and hence felt they were not using FHT enough, despite using it appropriately. These perceptions were exacerbated by the early technical problems. Lack of time spent exploring the platform, either due to competing priorities or a reluctance to engage, meant these pre-conceptions were not challenged. Some interviewees wondered if there was a ‘better way’ to use it, as they felt the way they were interacting with the platform was too simple. Practices who were part of the co-design team or whose staff members were part of the advisory group were more likely than other practices to persevere with FHT despite the initial problems.

Similarly, participant knowledge of clinical topics influenced their use of FHT. Where a recommendation was too simple and general, use tended to be limited. Where a recommendation suggested something that the clinician did not have experience in or agree with, use tended to be limited (Table 3, Quotes 14&15). Where recommendations provided an easy-to-follow suggestion, with direct and accessible links to clinical guidelines and resources, use increased.

## Discussion

The intention of this project was to test and improve FHT and its supporting implementation strategies, with a view to preparing FHT both for a randomised controlled trial and for large-scale implementation. This project demonstrated that FHT has the potential to improve the identification and management of chronic disease, such as CKD, and improve clinical performance in general practice. Clinicians reported that they became more aware of CKD and were more likely to act in line with CKD clinical guidelines after using FHT. Key co-design features of FHT [11], including the repeated delivery of guideline concordant recommendations underpinned by algorithms drawing data from the EMR to ensure specificity to individual patients, enhanced clinician trust and confidence in FHT.

Hak’s Maturity Staging Model [29] provides a framework to understand readiness for implementation of software tools. Using this lens, the FHT tool is situated at Stage 3 of maturity (Design + Intelligence + Choice), having successfully navigated the co-design, build and use of the tool in a real-world setting. The simplicity of the functionality and display of information influenced use of the tool, with recommendations most likely to be acted on when presented in clear, unambiguous language and simple to enact. FHT did not require additional implementation support or practice change to be used effectively. However, in keeping with Ivers’ 2012 [5] hypothesis, complex recommendations requiring interpretation or further investigation and recommendations with more complex clinical action were associated with negative perceptions of FHT and increased the likelihood that clinicians would disengage. Whilst explicit links to relevant guidelines were provided, these also needed to be simple, familiar, and accessible to facilitate engagement with and actioning of recommendations. With this in mind, and with the variability of practice structure, available time, and staff experience, more robust implementation strategies are required to support the further maturation of FHT to achieve Stage 4 (Design + Intelligence + Choice + Implementation). Such strategies need to combine improvements in the technology itself, including improvements to the user interface and logic flow, with techniques to actively engage practice staff with the clinical decision support recommendations.

Strategies to support implementation aimed to target key areas identified in previous research, including increasing connection or ownership of the tool [30–33], supportive resourcing and incentivisation, providing active audit and feedback, and providing practice-specific flexibility in the intervention [34]. 

Engagement and ownership can normalise (and increase) use of a clinical decision support tool [35]. Our findings supported this, with practices who expressed a sense of connection or ‘ownership’ of FHT and had available time to prioritise the tool being more likely to use FHT more extensively throughout the implementation period and more likely to continue using post-study. Rapid transition from ‘activation of software’ to ‘active use of software’, the iterative change to FHT after feedback provided by clinicians and the capacity of staff to engage with FHT all influenced connection, ownership and engagement with the tool, despite the COVID-19 pandemic further amplifying the challenging implementation environment in general practice [34]. 

Initially, in keeping with previous clinical decision support system implementation evaluations [30], we found technical barriers negatively impacted staff engagement. However, once the technical issues were resolved, clinicians who used the POC prompt reported that it improved their practice. Specifically, the prompt and its attached recommendations were perceived to be complementary to the practices’ existing workflow and were clinically meaningful. Whilst flexibility and adaptability have been identified as key factors in successful implementation of a new software [36, 37], this research indicated that much of FHT’s success resulted from being absorbed into existing workflows both within and in preparation for the consultation with the patient. It is interesting that both GPs and GPNs reported using FHT during consultations when previous research [38] has indicated that a clinical decision support tool used outside consultation was more likely to fit with general practice workflow and be perceived as useful.

A lack of compatibility with existing workflow, exacerbated by insufficient additional time and resources, resulted in the dashboard component of FHT being used less frequently, consistent with findings from Hespe [35], and in contrast to the conclusions of a systematic review by Chima [38]. The dashboard required additional action outside the usual workflow, with the need to access a specific URL and enter unique login. Language used to describe this component created associations with performance monitoring, something less favourably received in this cohort of clinicians. Previous studies [5, 30] have encountered similar difficulties in encouraging clinicians to seek out a dashboard and have reflected that a ‘push’ strategy (where information is actively provided to clinicians, including via a prompt) works more effectively than ‘pull’ strategy that attempts to lure them in using performance data. Whilst the intention was to develop FHT to provide benchmarking functionality to provide a lure for clinicians, technological challenges and practice reluctance has resulted in this component being removed.

These findings are being used to inform further development of FHT, including development of guidelines for use of language and links to resources for any new FHT recommendations, implementation materials and support for practices at the point of onboarding, broader access within the practice upon installation (i.e., not restricting access to one or two machines in the practice) and ensuring that recommendations developed for FHT are relevant and actionable within general practice. Further strategies to encourage future use of the dashboard included a rebrand as a ‘portal’ to resources and tools; adding a link from the POC; and refining login processes to use auto-login on a recognised machine.

### Limitations

General practice is a high pressure, resource-poor environment, in which new initiatives are difficult to implement. Although the practices were recruited to this project in late 2019, with an intended February 2020 commencement, implementation coincided with the beginning of the COVID-19 pandemic in Australia and continued through the numerous lockdowns experienced across Victoria. Significant resources in each practice were deployed to address COVID-19 protocols and FHT was not a priority among the daily work tasks. Variations in the planned evaluation approach did not have a significant impact on the project, however it did result in an absence of patient voice from the project. The planned patient interviews were to have provided the patient perspective on how clinician use of the FHT tool sat within the consultation, any barriers or facilitators they perceived to the provision of care, and suggestions for improvement. Independent verification of clinical performance improvement would enhance this evaluation and provide greater understanding of the potential of FHT to improve patient outcomes. Similarly, a further analysis of factors impacting implementation with a larger group of practices is required to determine if the observed effects are unique to early adopters or factors that can be flexibly adapted across a diversity of practices.

### Future work

FHT is a clinical decision support platform that can provide accessible translation of clinical guidelines into individualised recommendations for patient care. Opportunities for future study include the expansion of FHT to cover different health conditions beyond chronic disease, and the expansion of the technology to capture and present information in more interactive ways. Specific areas for attention include but are not limited to: review of the differences in the way the FHT coding works across EMR systems, including potential expansion to integrate with other EMR systems; in-depth analysis of usability and acceptability of the technology utilising frameworks designed to assess use of technology; examination of volume and prioritisation of clinical recommendations (i.e. is there a limit to how many different conditions can be captured and displayed to clinicians before they disengage); and patient perspectives on the place of FHT in clinical consultations.

## Conclusions

This evaluation aimed to examine the barriers and facilitators to implementing a new technological intervention to improve chronic kidney disease management in primary care practice. It is challenging to implement a technological intervention in primary care, and more so in the early stages of the COVID-19 pandemic. Flexible work arrangements (including working from home and alternate workspaces), the rapid uptake of telehealth, and a change in the presenting profile of patients when pandemic wariness was at its highest contributed to this implementation being unexpectedly complex. Despite this, the evaluation identified that FHT can be implemented successfully in general practice in Victoria. Changes were made to the software and implementation approach during and after this evaluation to increase accessibility and usability. Findings from this study have been used to inform a randomised controlled trial exploring the effectiveness of FHT in managing CKD and identifying patients at-risk of cancer and will support future implementation in a broader range of practices and of a wider range of conditions, including type two diabetes, cardiovascular disease, and risk of undiagnosed cancer.

### Electronic supplementary material

Below is the link to the electronic supplementary material.

Supplementary Material 1

Supplementary Material 2

## Data Availability

The datasets generated and/or analysed during the current study are not publicly available due to the size of the dataset and the nature of the information but are available from the corresponding author on reasonable request.

## Abbreviations

ACEI: Angiotensin converting enzyme inhibitors
ACR: Albumin-to-creatinine ratio
ARB: Angiotensin receptor blockers
BP: Blood pressure
CKD: Chronic kidney disease
CP-FIT: Clinical Performance-Feedback Intervention Theory
CVD: Cardiovascular disease
eGFR: Estimated Glomerular Filtration Rate
FHT: Future Health Today
GP: General practitioner
GPM: General practice manager
GPN: General practice nurse
LDL: Low density lipoprotein
POC: Point of care
TG: Triglyceride

## References

[1] Australian Institute of Health and Welfare Chronic Conditions and Multimorbidity. Australian Insitute of Health and Welfare. 2022 https://www.aihw.gov.au/reports/australias-health/chronic-conditions-and-multimorbidity Accessed 3 May 2024.

[2] Khanam M, Kitsos A, Stankovich J, Castelino R, Jose M, Kinsman L (2019). Chronic kidney disease monitoring in Australian general practice. Australian J Gen Practitioners.

[3] Australian Institute of Health and Welfare Chronic Kidney Disease: Australian Facts. Australian Insitute of Health and Welfare. 2022 https://www.aihw.gov.au/reports/chronic-kidney-disease/chronic-kidney-disease/contents/summary Accessed 3 May 2024.

[4] Kidney Health Australia. Chronic kidney disease (CKD) management in primary care. Kidney Health Australia 2020 https://assets.kidney.org.au/resources/CKD-Management-in-Primary-Care_handbook_2020.1.pdf Accessed 3 May 2024.

[5] Ivers N, Jamtvedt G, Flottorp S, Young JM, Odgaard-Jensen J, French SD et al. Audit and feedback: effects on professional practice and healthcare outcomes. Cochrane Database Syst Reviews. 2012;(6). 10.1002/14651858.CD000259.pub3 PMC11338587 22696318

[6] Johnson M, May C. Promoting professional behaviour change in healthcare: what interventions work, and why? A theory-led overview of systematic reviews. BMJ open. 2015;5. 10.1136/bmjopen-2015-008592 PMC4593167 26423853

[7] Carroll J, Pulver G, Dickinson M, Pace W, Vassalotti J, Kimminau K et al. Effect of 2 clinical decision support strategies on chronic kidney disease outcomes in primary care - a cluster randomised trial. JAMA Open Netw. 2018;1. 10.1001/jamanetworkopen.2018.3377 PMC6324427 30646261

[8] Pefanis A, Botlero R, Langham R, CL N (2018). eMAP:CKD: electronic diagnosis and management assistance to primary care in chronic kidney disease. Nephrol Dialysis Transplantation.

[9] Jones J, Lumsden N, Simons K, Fernando S, Neil C, Manski-Nankervis J et al. Detection and management of chronic kidney disease and diabetes with e-technology based intervention: analysis of the chronic disease early detection and improved management in PrimAry Care projecT (CD IMPACT). Nephrol Dialysis Transplantation 2019;34.

[10] Chen W, O’Bryan CM, Gorham G, Howard K, Balasubramanya B, Coffey P (2022). Barriers and enablers to implementing and using clinical decision support systems for chronic diseases: a qualitative systematic review and meta-aggregation. Implement Sci Commun.

[11] Hunter B, Biezen R, Alexander K, Lumsden N, Hallinan C, Wood A (2020). Future Health today: codesign of an electronic chronic disease quality improvement tool for use in general practice using a service design approach. BMJ open.

[12] MedicalDirector, Clinical. May https://www.medicaldirector.com/ Accessed 3 2024.

[13] BestPractice. Premier https://bpsoftware.net/ Accessed 3 May 2024.

[14] ZedMed. May https://www.zedmed.com.au/ Accessed 3 2024.

[15] Hunter B, Alexander K, Biezen R, Hallinan CM, Wood A, Nelson C et al. The development of future health today: piloting a new platform for identification and management of chronic disease in general practice. Aust J Prim Health. 2022. 10.1071/PY22022 36318973

[16] Chima S, Martinez-Gutierrez J, Hunter B, Manski-Nankervis J-A, Emery J (2022). Optimization of a Quality Improvement Tool for Cancer diagnosis in primary care: qualitative study. JMIR Form Res.

[17] Greenwood D, Levin M, Reason P, Bradbury H (2001). Pragmatic action research and the struggle to transform universities into learning communities. Handbook of action research.

[18] Webb C (1989). Action research: philosophy, methods and personal experiences. J Adv Nurs.

[19] Meyer J (2000). Qualitative research in health care. Using qualitative methods in health related action research. BMJ.

[20] Greenwood DJ, Levin M. Reform of the social sciences, and of universities through action research. In: Denzin NK, Lincoln YS, editors. The SAGE Handbook of Qualitative Research, 3rd Edition. Thousand Oaks: Sage Publications Ltd; 2005.

[21] Hart E, Bond M (1995). Action Research for Health and Social Care, A Guide to practice.

[22] Pawson R, Tilley N (1997). Realistic evaluation.

[23] Miller WL, Crabtree B, Denzin NK, Lincoln YS (2005). Clinical Research. The SAGE handbook of qualitative research 3rd Edition.

[24] Soós M, Temple-Smith M, Gunn J, Johnston-Ata’Ata K, Pirotta M (2010). Establishing the Victorian Primary Care Practice Based Research Network. Aust Fam Physician.

[25] VicREN Accessed 3 May 2024: https://medicine.unimelb.edu.au/school-structure/general-practice-and-primary-care/engagement/primary-care-community

[26] Nelson J. Using conceptual depth criteria: addressing the challenge of reaching saturation in qualitative research. 2017;17(5):554–70.

[27] NVivo. (Version 12) https://www.qsrinternational.com/nvivo-qualitative-data-analysis-software/home. QSR International Pty Ltd; 2018 Accessed 3 May 2024.

[28] Brown B, Gude WT, Blakeman T, van der Veer SN, Ivers N, Francis JJ (2019). Clinical performance feedback intervention theory (CP-FIT): a new theory for designing, implementing, and evaluating feedback in health care based on a systematic review and meta-synthesis of qualitative research. Implement Sci.

[29] Hak F, Guimarães T, Santos M (2022). Towards effective clinical decision support systems: a systematic review. PLoS ONE.

[30] Patel B, Usherwood T, Harris M, Patel A, Panaretto K, Zwar N (2018). What drives adoption of a computerised, multifaceted quality improvement intervention for cardiovascular disease management in primary healthcare settings? A mixed methods analysis using normalisation process theory. Implement Sci.

[31] Orchard J, Li J, Gallagher R, Freedman B, Lowres N, Neubeck L (2019). Uptake of a primary care atrial fibrillation screening program (AF-SMART): a realist evaluation of implementation in metropolitan and rural general practice. BMC Fam Pract.

[32] Jeffries M, Phipps DL, Howard RL, Avery AJ, Rodgers S, Ashcroft DM (2017). Understanding the implementation and adoption of a technological intervention to improve medication safety in primary care: a realist evaluation. BMC Health Serv Res.

[33] Bonawitz K, Wetmore M, Heisler M, Dalton VK, Damschroder LJ, Forman J (2020). Champions in context: which attributes matter for change efforts in healthcare?. Implement Sci.

[34] Ivers NM, Sales A, Colquhoun H, Michie S, Foy R, Francis JJ (2014). No more ‘business as usual’ with audit and feedback interventions: towards an agenda for a reinvigorated intervention. Implement Sci.

[35] Hespe C, Rychetnik L, Peiris D, Harris M (2018). Informing implementation of quality improvement in Australian primary care. BMC Health Serv Res.

[36] Grant A, Dreischulte T, Guthrie B (2017). Process evaluation of the Data-driven quality improvement in primary care (DQIP) trial: case study evaluation of adoption and maintenance of a complex intervention to reduce high-risk primary care prescribing. BMJ Open.

[37] Brehaut JC, Colquhoun HL, Eva KW, Carroll K, Sales A, Michie S (2016). Practice feedback interventions: 15 suggestions for optimizing effectiveness. Ann Intern Med.

[38] Chima S, Reece JC, Milley K, Milton S, McIntosh JG, Emery JD (2019). Decision support tools to improve cancer diagnostic decision making in primary care: a systematic review. Br J Gen Pract.

