# Supplementary material for: Optimising a clinical decision support tool to improve chronic kidney disease management in general practice
Source: BMC Prim Care. 2024 Jun 19;25:220. doi: 10.1186/s12875-024-02470-w (PMC11186183; doi:10.1186/s12875-024-02470-w)
Supplement: Supplementary file 1 — Supplementary Material 1 [file 12875_2024_2470_MOESM1_ESM.docx]

Figure S1. FHT Software Components – Point of Care Prompt


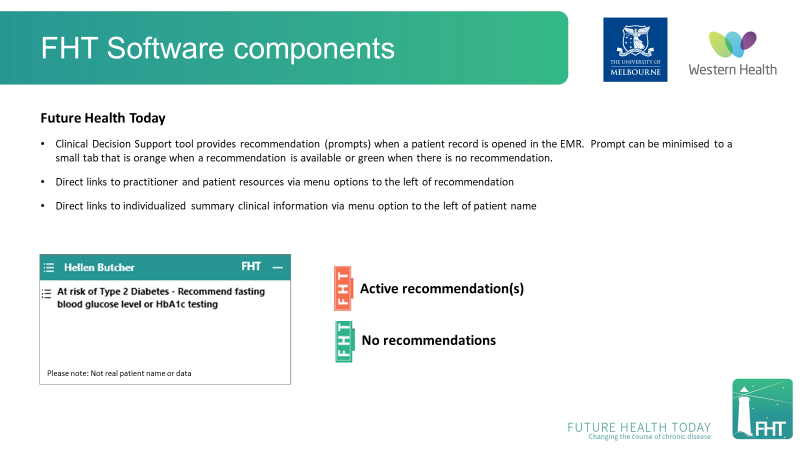


Figure S2. FHT Software Components: Dashboard


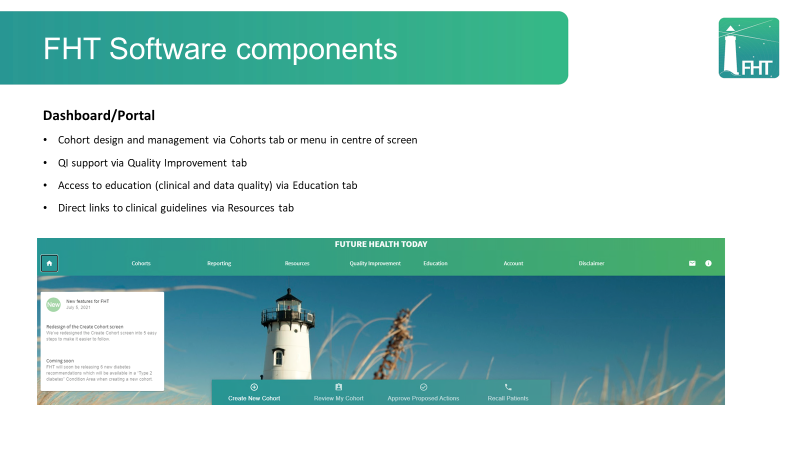


Figure S3. FHT Software Components: Cohort Creation Tool


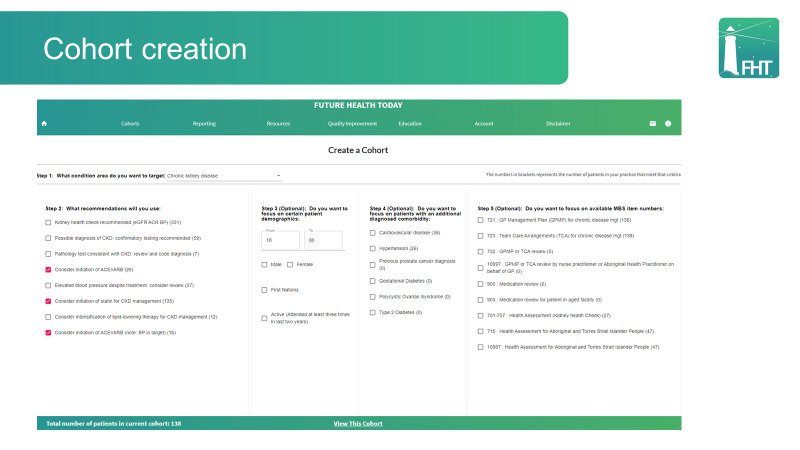


Figure S4. FHT Software Components: Cohort Review Page


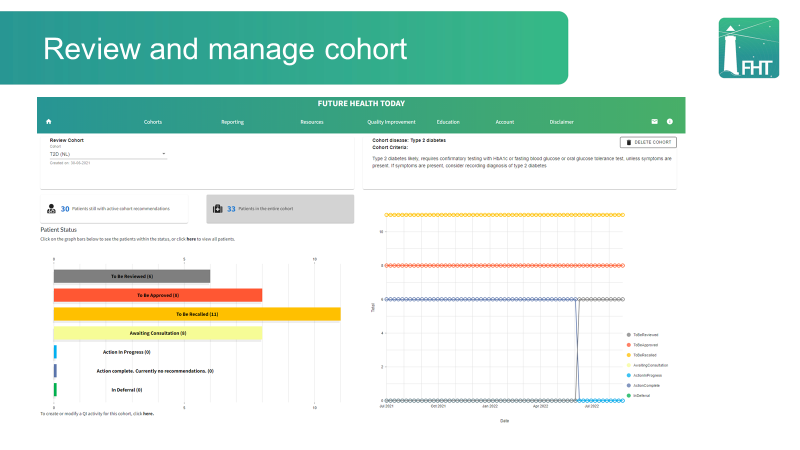


Please note that in this screenshot, the bar graph on the left displays the number of patients in the following categories: To be reviewed, To be approved (for recall or deferral), To be recalled, Awaiting consultation, Action in progress, Action complete (currently no recommendations) and In deferral. The number of patients in each category is provided in brackets beside the bar label. The line graph on the right maps change in number of patients in these categories over time.
