# Supplementary material for: Optimising a clinical decision support tool to improve chronic kidney disease management in general practice
Source: BMC Prim Care. 2024 Jun 19;25:220. doi: 10.1186/s12875-024-02470-w (PMC11186183; doi:10.1186/s12875-024-02470-w)
Supplement: Supplementary file 2 — Supplementary Material 2 [file 12875_2024_2470_MOESM2_ESM.docx]

**Abbreviated ‘Short Check in’ Interview – Interview #1**

*Anticipated length:* 5-10 mins

*Anticipated participants:* Practice Champion & participating PNs (if not champion) & GPs

*When:* 1 month post installation

*Commence call with questions around use:*

- Have you used FHT? What components?
- Do you find it easy to use? Can you get to the pages you need to get to? Does it make sense?
- Are you able to do the things you need to do using FHT?
- Is there anything else you would like it to do?
- Do the components of Future Health Today fit into the workflow of
  - the consultation
  - the practice
- Is there additional information that you would require to carry out your tasks in chronic disease management that was not included in Future Health Today?
- How would you change Future Health Today, if at all?

*Ask about PoC – location, colour, appearing, minimising

*Ask about ‘news’ – where would they like to see it located or how would they like to receive it

***Very optional extra – not necessary unless there are issues or concerns with any particular area***

Then ask if they would like to turn it on and talk you through the component they are using at the moment.

1. Speak aloud any words in their mind as they complete the tasks, which may include:

- Generation of a list of patients with risk factors for chronic kidney disease
- Review of performance of practice in recording chronic kidney disease compared to other practices (using an audit and benchmarking portal)
- Recording of planned quality improvement activities in the platform
- Use of recall and patient monitoring systems
- Use of point of care clinical decision support

**Example interview script questions for practice staff – Interview #2 and subsequent as required**

*Anticipated length:* 15-20 mins

*Anticipated participants:* Practice Champion & participating PNs & GPs

*When:* 2-3 month post installation

*Note that interview schedule is only indicative and will be tailored based on the interviewee e.g. GP, practice nurse or practice manager, the stage of implementation and whether they have been interviewed previously.*

Hi, thanks for agreeing to share with us your experiences of using the Future Health Today technology in your practice.

Before we start, I’d just like to remind you that we are audio recording this session, but that the recordings will be stored securely and only used by the researchers. We will make sure that we remove identifying details as much as we can as well.

Do you have any questions before we start?

I’d like to start off by asking how you identified people at risk of, or with, chronic kidney disease prior to participating in Future Health today?

*Potential probing questions:*

- *Kidney Health Australia guidelines*
- *Electronic audit tools e.g. PenCAT*
- *What prompts you to discuss CKD risk or diagnosis with patients?*
- *Did you ever recall patients for this reason? How did you do that? What types of patients? Who did the recall?*

Since using Future Health Today, who do you think would use it in your practice?

*Potential probing questions:*

- *Was there anyone who you felt it should be used for but it wasn’t possible/appropriate?*
- *Why/why not?*
- *Did you feel you used it unnecessarily with anyone?*
- *Did you think the other nurses used it and/or wanted to use it?*
- *Did you feel the GPs were comfortable with you using it with patients?*
- *Did you discuss Future Health Today with others in your clinic?*
- *How well were your leadership team involved?*
- *Do you believe that others in the clinic are committed to using Future Health Today?*
- *What else could have been done?*
- *Was there any resistance? If so, what and who resisted?*

In your opinion, was Future Health Today effective?

*Potential probing questions:*

- *Did you feel that using Future Health Today increased the likelihood of appropriate diagnosis and management of chronic kidney disease in your patients?*
- *Prompt: How would you modify Future Health Today to make it more effective?*

Which of the implementation strategies did you find the most effective?

*Potential probing questions:*

- *Which were the most effective implementation strategies/least effective? Prompt: mention the specific methods in the design*
- *Why did you think that the method described was/wasn’t effective?*
- *How might you make that sustainable or change that in your clinic?*

Have you used the Future Health Today tool to identify and recall patients? If so, how did that go? If not, can you tell me why you haven’t used it?

*Potential probing questions:*

- *How did you feel using Future Health Today?*
- *How confident were you in your ability to use Future Health Today? What made/would make you feel more confident?*
- *Was there anything that you thought might have made it better to use?*
- *What might facilitate you using it as a regular tool?*
- *Did anything make you feel like you might not want to use it again?*
- *Was there anything that made it difficult to adopt?*
- *Why/why not? (all above)*

Have you used Future Health Today in a consultation to discuss chronic kidney disease targeted testing or management with a patient? If so, how did the consultation go?

*Potential probing questions:*

- *How did you feel using Future Health Today?*
- *How confident were you in your ability to use Future Health Today? What made/would make you feel more confident?*
- *Do you feel that you/the patient understood the information?*
- *Was there anything that you thought might have made it better to use?*
- *What might facilitate you using it as a regular tool?*
- *Did anything during the consultation make you feel like you might not want to use it again?*
- *Was there anything that made you feel that you would like to use Future Health Today in another*

*consultation?*

- *Was there anything that made it difficult to adopt?*
- *Why/why not? (all above)*

How did you find the strategies that we incorporated into the study to increase the use and ease

of use of Future Health Today?

*Potential probing questions:*

- *Did you notice them? If yes, was that a positive or negative thing?*
- *Were there any methods that were never used? Prompt: check implementation strategies as*

*necessary*

- *Would you suggest anything else that might be used/developed to increase the implementation of Future Health Today within the clinic system?*
- *Why/why not? (all above)*

Future Health Today can be used as part of a practice quality improvement activity. Did you use Future Health Today in this way?

*Potential probing questions:*

- *Can you tell me about how Future Health Today was used for quality improvement?*
- *What might facilitate the use of Future Health Today for quality improvement?*
- *Was there any additional features that would have made it easier to use in this way?*
- *Participation in QIPIP?*
- *Why/why not? (all above)*

Did you use Future Health Today as much since I last spoke to you or less or about the same?

*Potential probing questions:*

- *Is it easy to maintain or difficult? Why/why not?*
- *Did you remember to use Future Health Today?*
- *Do you think that you and your colleagues will use Future Health Today moving forward?*
- *Do you have any ideas about how Future Health Today might become more sustainable?*
- *What might facilitate you using it as a regular tool?*

What did you think of Future Health Today overall?

*Potential probing questions:*

- *Do you think it is useful for identifying people at risk of CKD, or who have CKD and might benefit from optimisation therapy?*
- *Did you agree with the recommendations?*
- *What do you think of the quality of the program – the design/look/features*
- *Do you think others in your clinic see the value in using Future Health Today routinely?*

Is there anything else that you would like to say about using Future Health Today?

Is there anything else you would like to say about the use of technologies like Future Health today in general practice?
